# Supplementary material for: Position error-free control of magnetic domain-wall devices via spin-orbit torque modulation
Source: Nat Commun. 2023 Nov 23;14:7648. doi: 10.1038/s41467-023-43468-9 (PMC10667336; doi:10.1038/s41467-023-43468-9)
Supplement: Supplementary file 1 — Supplementary Information [file 41467_2023_43468_MOESM1_ESM.pdf]

Supplementary Information

Position error-free control of magnetic domain-wall devices via spin-orbit torque modulation

Seong-Hyub Lee[1‡], Myeonghoe Kim[1‡], Hyun-Seok Whang[1‡], Yune-Seok Nam[1], Jung-Hyun Park[1], Kitae Kim[1], Minhwan Kim[1,2], Jiho Shin[1], Ji-Sung Yu[1], Jaesung Yoon[1], Jun-Young Chang[1,2], Duck-Ho Kim[2], and Sug-Bong Choe[1*]

[1]Department of Physics and Institute of Applied Physics, Seoul National University, Seoul, 08826, Republic of Korea

[2]Center for Spintronics, Korea Institute of Science and Technology, Seoul, 02792, Republic of Korea

Table of contents

I. Supplementary Notes 2

Supplementary Note 1. Opposite unidirectional motion with different value of L/w 2

Supplementary Note 2. Sample preparation 2

Supplementary Note 3. Measurement of SOT-modulated micro devices 2

Supplementary Note 4. Characterization of nano device 2

Supplementary Note 5. Generation of DMI by breaking inversion symmetry 2

Supplementary Note 6. Spin-orbit torque measurement 2

Supplementary Note 7. Independent control of SOT 2

II. Supplementary Figures 3

Supplementary Figure 1 Opposite unidirectional unlocking of domain walls. 3

Supplementary Figure 2 Plot of V_H with respect to H_z. 3

Supplementary Figure 3 Sequence of image process in micro scaled SOT-modulation device. 3

Supplementary Figure 4 Line profile of nanodevice. 4

Supplementary Figure 5 Plot of v_{DW} as a function of H_x. 4

Supplementary Figure 6 Illustration of experimental setup with microscope images. 4

Supplementary Figure 7 Measurement of SOT efficiency. 5

Supplementary Figure 8 Plot of ε as a function of H_x for diverse material structures. 5

Supplementary Figure 9 Plot of ε as a function of H_x for different values of t_{Pt}. 5

III. Supplementary References. 5

I. Supplementary Notes

Supplementary Note 1. Opposite unidirectional motion with different value of L/w

By contrast to the case for $L < w/2$ in Fig. 2, here we show the opposite unidirectional motion of domain wall in the case for $L > w/2$ as seen in Supplementary Fig. 1a, where w ($= 100$ nm) is the wire width, and L ($= 40$ nm) is the width of the central area. Supplementary Fig. 1b illustrates snapshots of a micromagnetic simulation for when a domain wall is pushed toward modulation boundary, from right to left. Here, the black and white areas correspond to the down and up domains, respectively, with the domain wall in between. In the present situation, a negative current ($I_c < 0$) generates a negative driving force that pushes the domain wall to the left. As the domain wall travels across the boundary, the domain wall stops under the reverse force from the central area of the thickness modulation. It is worth noting that when the domain wall stops at the equilibrium position, the domain wall lies less on the central area owing to its unequal widths. If one reverses the current polarity at this moment, the driving forces are also reversed to push the domain wall away from the modulation boundary. Subsequently, owing to the unequal widths, the domain wall is pushed more away from the central-area side, as shown in Fig. 1c.

Supplementary Note 2. Sample preparation

For this study, all the samples were prepared on 525 μm Si/100 nm SiO_2 substrates by DC magnetron sputtering, where the base pressure is 3×10^{-8} torr. To minimize intermixing and nucleation sites, sputtering plasma was maintained at ~ 20 W (power density ~ 0.987 W/cm^2, deposition rate ~ 0.02 nm/s) at the 2 mtorr Ar atmosphere. Ta seed layer was used for enhancing adhesion of the Pt layer to substrate. Supplementary Fig. 2 shows the out-of-plane hysteresis loop of 5 nm Ta/2.5 nm Pt/0.3 nm Pd/0.4 nm Co/ 1.5 nm Pt sample which shows perpendicular magnetic anisotropy.

Supplementary Note 3. Measurement of SOT-modulated micro devices

On top of the Ta/Pt/Pd/Co/Pt structure, SOT modulation boundaries are built through the photo (e-beam) lithography, Pt deposition and lift-off process for micro (nano) scaled devices. Micro devices are measured by full-field polar MOKE microscopy, where field of view is 200×150 μm and time resolution is ~ 30 ms. After the magnetization is saturated by external magnetic field, background images are captured *N* (=100) times (Supplementary Fig. 3a) and averaged (Supplementary Fig. 3b) to suppress the shot noise. After domain wall is positioned at the modulation boundary, also images are captured (Supplementary Fig. 3c) and averaged (Supplementary Fig. 3d). For the high contrast between up and down domain wall and sharp edge of wire and modulations, each images are subtracted to back ground image (Supplementary Fig. 3e).

Supplementary Note 4. Characterization of nano device

Topography image of nano device is acquired by atomic force microscopy (Supplementary Fig. 4). Supplementary Fig. 4b is line profile of the image, where the sharp edge of modulation boundary with a height of ~ 2 nm and precise alignment between nanowire and SOT modulation are shown.

Supplementary Note 5. Generation of DMI by breaking inversion symmetry

Since the DMI generated by structural inversion asymmetry, by changing the stacking order of Pd/Co in Ta/Pt/Pd/Co/Pt structure, one can change the sign of DMI (Supplementary Fig. 5). Based on the domain wall velocity measurement method [1], domain wall velocity, v, with respect to H_x is plotted. Pt/Co/Pt film shows negligible magnitude of DMI, where the position of symmetry axis ($H_{sym} = -H_{DMI}$, vertical black line) becomes zero. On the other hand, by introducing Pd layer on top (bottom) of the Co layer, negative (positive) sign of DMI is measured as red (blue) vertical line at the Supplementary Fig. 6.

Supplementary Note 6. Spin-orbit torque measurement

To measure the spin-orbit torque (SOT) using domain wall (DW), we fabricated a micro wire with Ta/Au electrodes using the photolithography, ion milling, and lift-off process, as depicted in Supplementary Fig. 6. By applying current I, the spins σ_y in the *y* direction are injected to domain wall (DW), resulting in effective field $H_{z,eff}$. The effective field is described as $H_{z,eff} \propto Im_x \times \sigma_y$. Hence, the quantification of SOT can be achieved by measuring the DW depinning field, H^*_{dep}, using the following sequence [2,3]. To saturate the domain, large out-of-plane magnetic field H_z is applied. Then, DW is nucleated by the writing pulse (inset of Supplementary Fig. 6). While sweeping H_z, magneto-optical Kerr effect (MOKE) signal is measured. As shown in Supplementary Fig. 7a, H_{dep} is measured by the abrupt increase in MOKE signal and also, by applying I, H_{dep} is varied by $H_{z,eff}$. The measured depinning field H^*_{dep} described as $H^*_{dep} = H_{dep} - H_{z,eff} = H_{dep} - \varepsilon \times I$, where ε is SOT efficiency (Supplementary Fig. 7b). Finally, ε with respect to in-plane magnetic field H_x is depicted in Supplementary Fig. 7c. Note that ε becomes zero when H_x equals $-H_{DMI}$ due to the presence of Dzyaloshinskii-Moriya interaction (DMI)-induced effective field, H_{DMI}.

Supplementary Note 7. Independent control of SOT

The main idea of this study is modulating sign of spin-orbit torque, however this cannot be established without preserving DMI. Supplementary Fig. 8 shows that not only SOT is changed but DMI also affected by the changed material structure. As a result, all the zero-field SOT efficiencies, ε_0, show positive values. However, as shown in supplementary Fig. 9, H_{DMI} keeps invariant for all the different t_{Pt} values.

II. Supplementary Figures

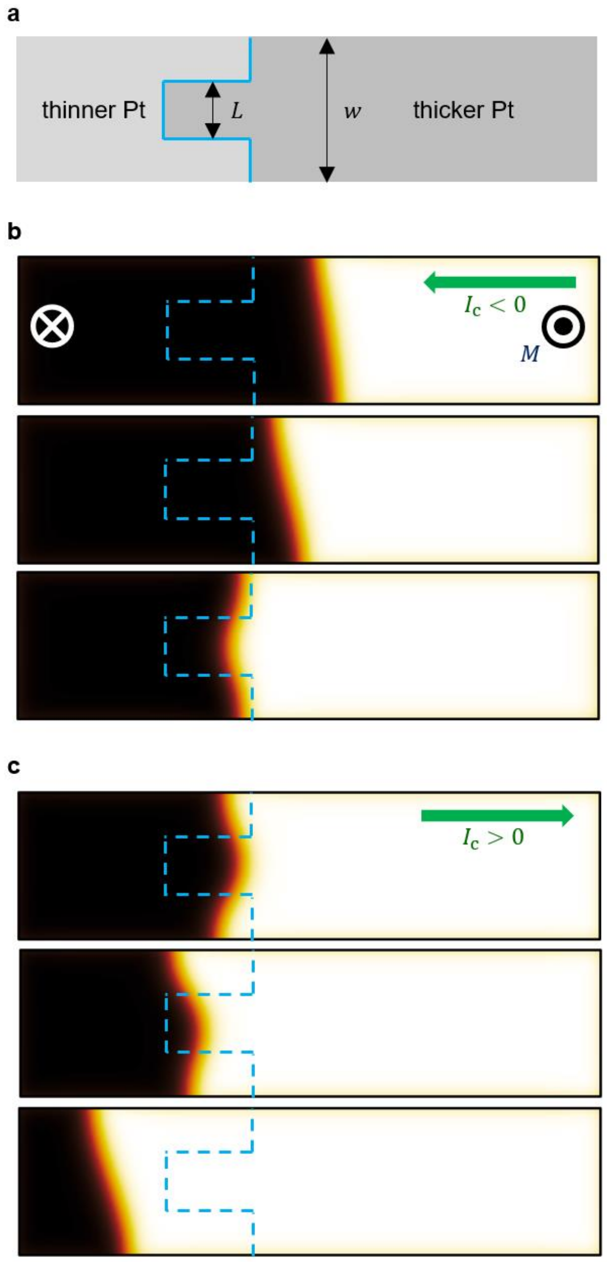

Supplementary Figure 1 Opposite unidirectional unlocking of domain walls. a Geometry of asymmetric modulation boundary (solid blue line) between the thicker (dark gray) and thinner (light gray) Pt areas, where w and L are the total wire width (100 nm) and central-area width (40 nm), respectively. **b, c** Sequential snapshot images of the micromagnetic simulation for the situations for domain-wall locking (**b**) and unlocking (**c**) owing to the negative and positive directions of injected current, respectively. White and black areas correspond to the domains magnetized either out of ◉ or into (⊗) the plane, respectively.

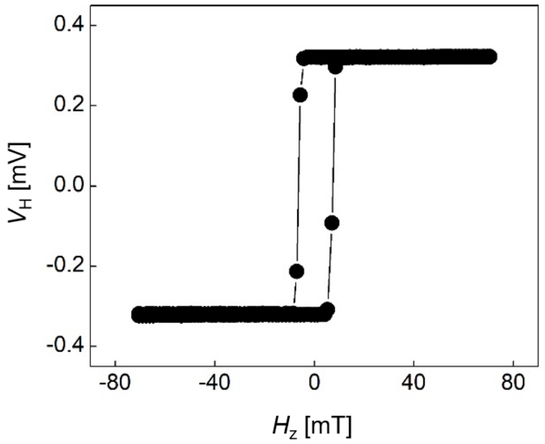

Supplementary Figure 2 Plot of V_H with respect to H_z. Hall voltage V_H of 5 nm Ta/2.5 nm Pt/0.3 nm Pd/0.4 nm Co/1.5 nm Pt sample is measured by sweeping out-of-plane magnetic field H_z

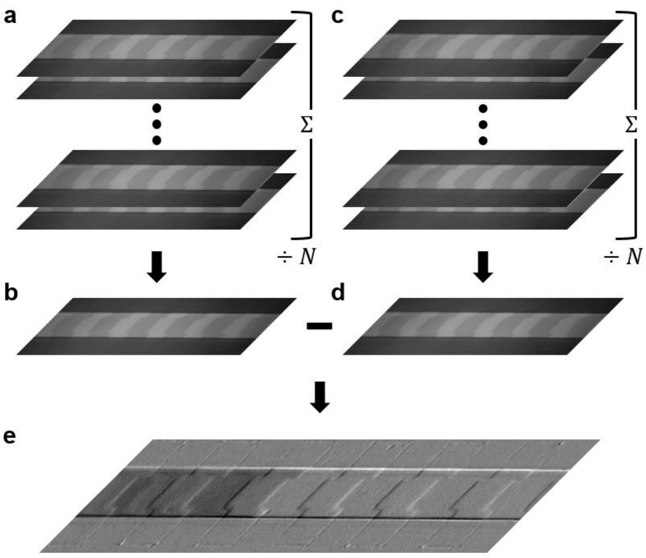

Supplementary Figure 3 Sequence of image process in micro scaled SOT-modulation device. a, c Background images acquired by MOKE microscopy are repeatedly N $(= 100)$ times captured without domain wall (**a**) and with domain wall (**c**). **b, d** Averaged images are acquired without domain wall (**b**) and with domain wall (**d**). **e**. High contrast image is taken by subtracting each other.

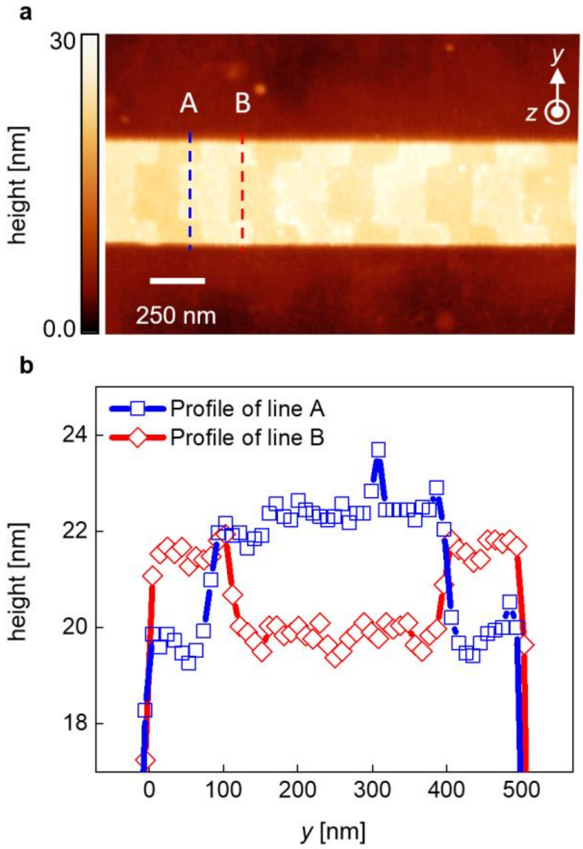

Supplementary Figure 4 Line profile of nanodevice. a Atomic force microscope image of the nano device. **b** Line profile of image (**a**). The figure shows ~ 2 nm of additional Pt layer thickness, sharp edge of modulation boundary and precise alignment between the modulation and nanowire.

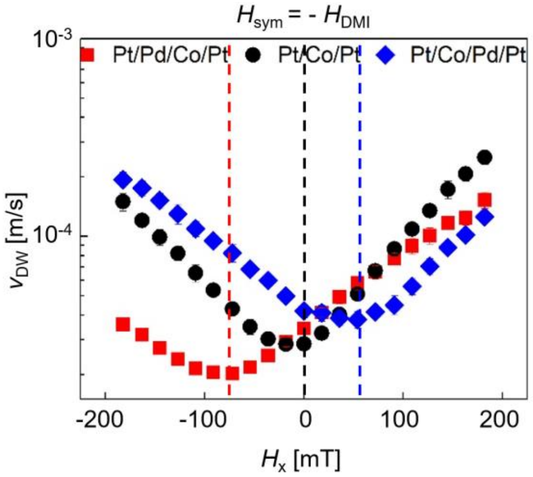

Supplementary Figure 5 Plot of v_{DW} as a function of H_{x}. Solid symbols indicate experimental data. The vertical dashed lines are depicted at $H_{\mathrm{sym}} = -H_{\mathrm{DMI}}$ for each sample.

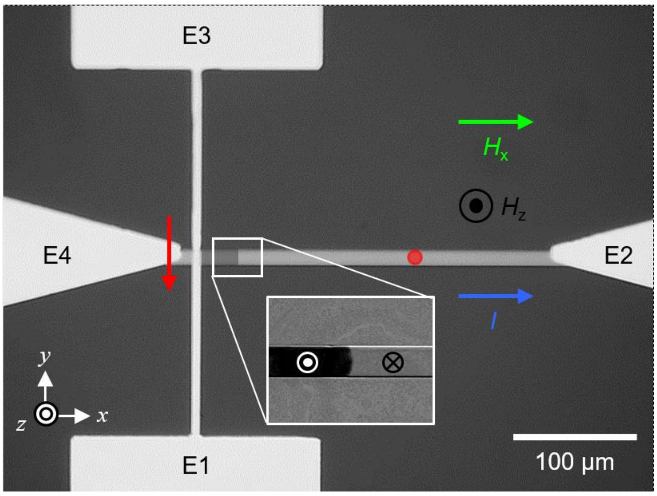

Supplementary Figure 6 Illustration of experimental setup with microscope images. Ta/Au electrodes (E1~E4) electrically contact with ferromagnetic wire. Domain wall nucleation pulse, H_x, I and H_z is drawn as red, green, blue arrow and symbol (◉), respectively. The area of domain wall nucleation and beam spot of laser MOKE is shown by gray region and red circle on a ferromagnetic wire. Inset shows nucleated domain wall, where the symbols ◎ and ⊗ indicate the direction of magnetization.

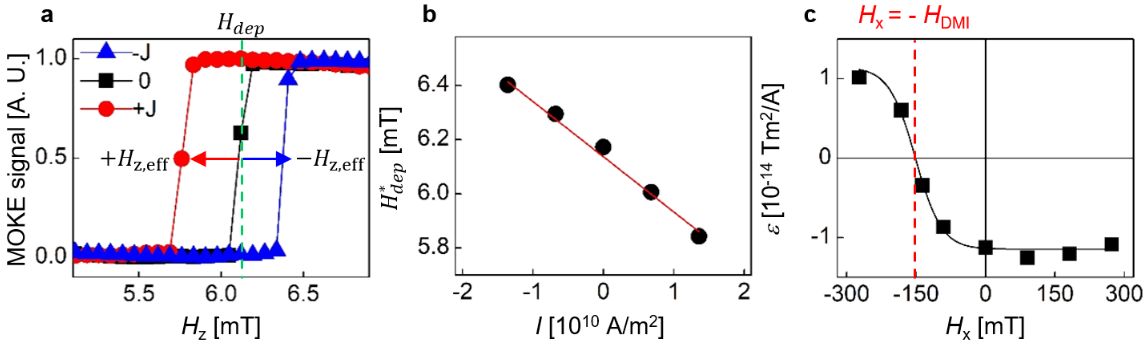

Supplementary Figure 7 Measurement of SOT efficiency. Solid symbols and curved lines indicate experimental data and best fitting to guide eye. **a** Plot of MOKE signal with respect to H_z, under the $-I$ (red), 0 (black) and $+I$ (blue) with $I = 1.36 \times 10^{10}$ A/m^2. H^*_{dep} and $H_{z,\mathrm{eff}}$ is depicted as green line and arrows. **b** H^*_{dep} with respect to I. The red line shows the best linear fit. **c** ε with respect to H_x is plotted. The vertical red line depict the axis $H_x = -H_{\mathrm{DMI}}$

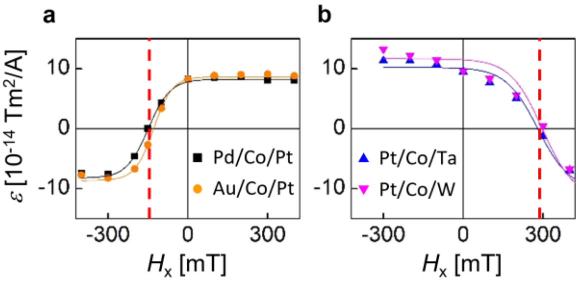

Supplementary Figure 8 Plot of ε as a function of H_x for diverse material structures. Solid symbols and curved lines indicate experimental data and best fitting to guide eye. The vertical red line depict the axis $H_x = -H_{\mathrm{DMI}}$, where ε becomes zero. **a** Data for X/Co/Pt where X is Pd and Au. **b** Data for Pt/Co/Y, where Y is Ta and W

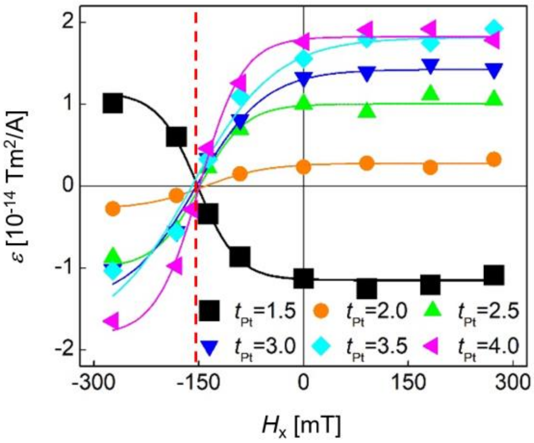

Supplementary Figure 9 Plot of ε as a function of H_x for different values of t_{Pt}. Solid symbols and curved lines indicate experimental data and best fitting to guide eye. The vertical red line depict the axis $H_x = -H_{\mathrm{DMI}}$, where $\varepsilon = 0$. The detailed structure of samples are 5.0 nm Ta/2.5 nm Pt/0.3 nm Pd/0.4 nm Co/t_{Pt} Pt.

III. Supplementary References.

1. Je, S.-G, Kim, D.-H, Yoo, S.-C, Min, B.-C, Lee, K.-J & Choe, S.-B. Asymmetric magnetic domain-wall motion by Dzyaloshinskii-Moriya interaction. *Phys. Rev. B* **88**, 214401 (2013).

2. Haazen, P. P. J., Murè, E., Franken, J. H., Lavrijsen, R., Swagten, H. J. M. & Koopmans, B. Domain wall depinning governed by the spin Hall effect. *Nat. Mater.* **12**, 299-303 (2013).

3. Emori, S., Martinez, E., Lee, K.-J., Lee, H.-W., Bauer, U., Ann, S.-M., Agrawal, P., Bono, D. C. & Beach G. S. D. Spin Hall torque magnetometry of Dzyaloshinskii domain walls. *Phys. Rev. B* **90**, 184427 (2014).
