# Supplementary material for: Position error-free control of magnetic domain-wall devices via spin-orbit torque modulation
Source: Nat Commun. 2023 Nov 23;14:7648. doi: 10.1038/s41467-023-43468-9 (PMC10667336; doi:10.1038/s41467-023-43468-9)
Supplement: Supplementary file 3 — Description of Additional Supplementary Files [file 41467_2023_43468_MOESM3_ESM.pdf]

Description of Additional Supplementary Files

File name: Supplementary Movie 1

Description: Micromagnetic simulation for unidirectional unlocking of domain walls

File name: Supplementary Movie 2

Description: Micromagnetic simulation for opposite unidirectional unlocking of domain walls

File name: Supplementary Movie 3

Description: Real-time observation for unidirectional motion of domain wall in micro-scale devices. (Uploaded at the public repository https://zenodo.org/records/10065364)
