# Supplementary figures and images for: Position error-free control of magnetic domain-wall devices via spin-orbit torque modulation
Source: Nat Commun. 2023 Nov 23;14:7648. doi: 10.1038/s41467-023-43468-9 (PMC10667336; doi:10.1038/s41467-023-43468-9)

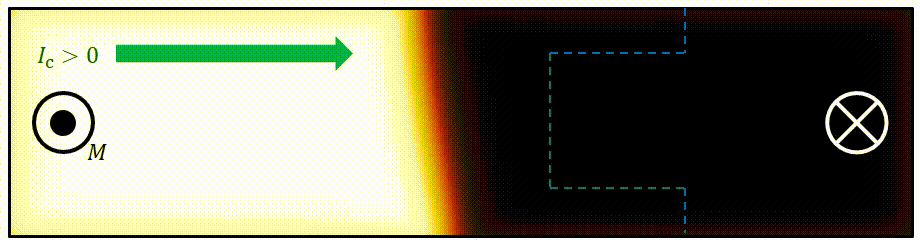

Supplement: Supplementary file 4 — Supplementary Movie 1 [file 41467_2023_43468_MOESM4_ESM.gif]

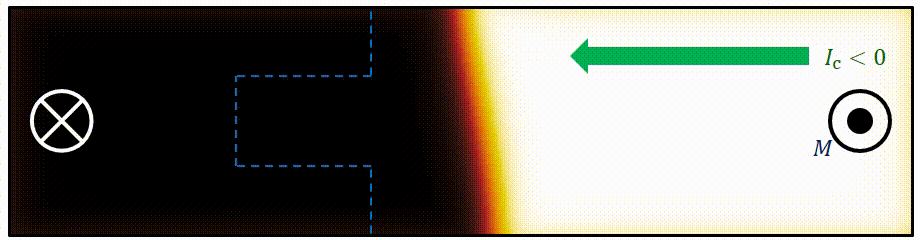

Supplement: Supplementary file 5 — Supplementary Movie 2 [file 41467_2023_43468_MOESM5_ESM.gif]

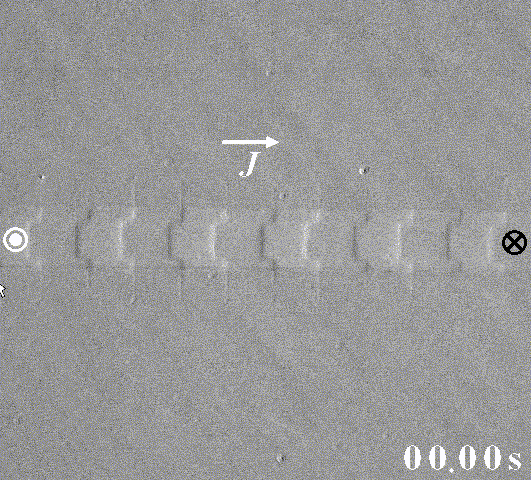

Supplement: Supplementary file 6 — Supplementary Movie 3 [file 41467_2023_43468_MOESM6_ESM.gif]
